# Dataset on analysis of quality of health and social insurance subscription in different socio-economic class of workers in selected areas in southwest Nigeria

**DOI:** 10.1016/j.dib.2018.10.135

**Published:** 2018-10-29

**Authors:** Titilope M. Dokunmu, Cynthia U. Adjekukor, David O. Oladejo, Emmanuel O. Amoo

**Affiliations:** a Department of Biochemistry, Covenant University, Ota, Nigeria; b Department of Demography and Social Statistics, Covenant University, Ota, Nigeria

## Abstract

National social health insurance scheme aims to improve the health of citizens and provide equal access to health care across different income classes. This empirical datasets describes quality of health, insurance subscription, awareness, health care coverage and benefits in different socio-economic class of workers in Ota and Lagos, Nigeria. The perception of individual׳s state of health and level of satisfaction of accessed health care are reported and opinions on ways to meet the health needs of workers in a developing country such as Nigeria.

**Specifications table**

**Table:** 

Subject area	Public health, Social science
More specific subject area	Social Health insurance, Health insurance policy
Type of data	Tables, figures, text file
How data was acquired	Questionnaires
Data format	Raw, Analysed
Experimental factors	Respondents were stratified into different socio-economic classes based on their income to determine rate of health insurance subscription and the effects on quality of health
Experimental features	Proportions, frequencies were used for correlation analysis of quality of health
Data source location	Ota; Ikorodu; Lekki (in Nigeria)
	7.9452°N, 4.7888°E, 6.6194°N, 3.5105°E, 6.4698°N, 3.5852°E
Data accessibility	Data are available within this article

**Value of the data**

• The data is valuable for improving health care and service delivery provided by health insurance schemes in Nigeria.
• The present data provides awareness for individuals on access to and benefits of social health insurance in Nigeria.
• The data shows health burdens and health care costs of different classes of workers.
• The data provides information for policy decision makers and government to improve awareness of existing schemes to impact and improve the overall health of all citizen of Nigeria.

## 1 Data

Disease prevalence is highest in low and middle income countries [1], [2]. Robust health care plays a major role in the sustainability and viability of a nation׳s social and economic growth [3]. This is a major reason for the idea of a National Health Insurance Scheme (NHIS) in Nigeria, which was first considered in 1962 [4] and its established operations in 2005 [5]. The NHIS was created to provide access to adequate and affordable health care for all Nigerians, but the scheme has been limited in achieving this due to declining revenue from crude oil exports previously used for public health funding [6]. Poor resources of low income earners and unemployment also limit individuals from benefiting [7]. In 2008, a World Bank survey reported that about 0.8% of the Nigerian population was covered by the NHIS [7], others access health schemes provided by their employers or parents, but out-of-pocket funding is more common [8], [9]. This dataset describes subscription rates of health insurance in different economic classes of workers, access to this social amenity, health status of those who subscribe and suggestions on ways to improve health insurance and health care delivery in Nigeria [10], [11], [12], [13].

## 2 Experimental design, materials and methods

Structure based questionnaires were designed according to standards to determine respondents’ age range, economic/occupational status based on income, health status, voluntary or employer health insurance subscription, monthly cost of care, medical coverage of the insurance, international health coverage and satisfaction of health care delivery in three socially distinct economic classes of Nigerian workers. The study protocol was approved by Covenant University Biological Sciences Research Ethics Committee (CUBIOSCREC) in 2016, with approval number CU/BIOSCRECU/BIO/2016/055.

### 2.1 Sampling

Healthy participants aged ≥15years voluntarily participated in the health survey. The respondents within working age group were sampled from three geographical locations in southwest Nigeria: Lekki and Ikorodu in Lagos state and Ota, Ogun state. Three distinct socio-economic classes were used, based on the occupational status of the respondents. A sample size of 201 was used with margin of error of 6.93, confidence level (*α*) of 95% and power of 0.9.

### 2.2 Analyses overview

The data were analyzed using the statistical software (SPSS) for descriptive statistics to determine correlation between the co-variants. Data is presented as bars, charts and table. The data was stratified based on valid responses in employed socio-economic class (*n* = 168) and all respondents (*n* = 201) with valid response.

### 2.3 Data presentation

**Fig. 1:**
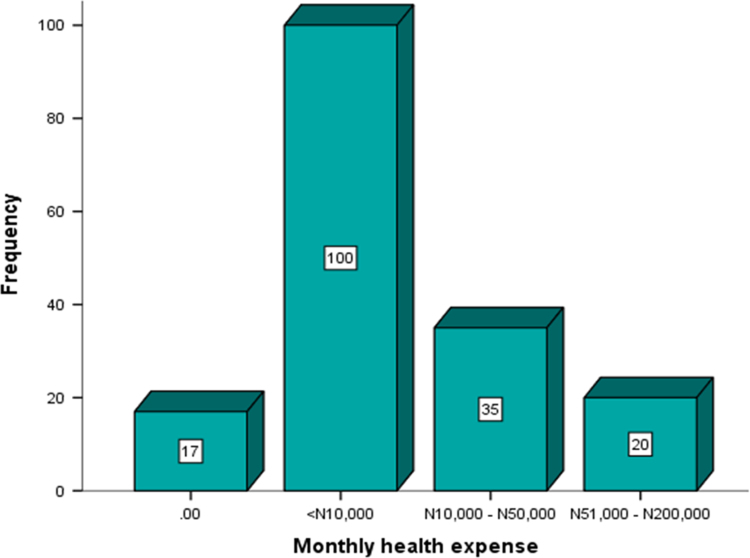
Average monthly medical cost reportedly incurred by all respondents.

**Fig. 2:**
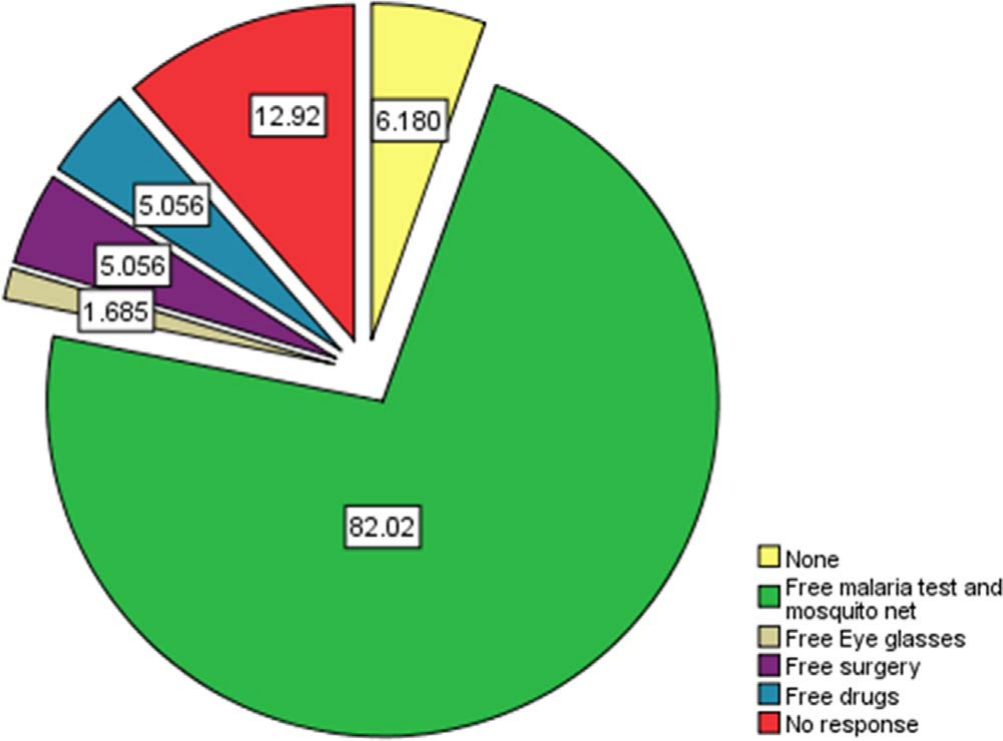
Summary of freely accessible government health care schemes available to citizens.

**Fig. 3:**
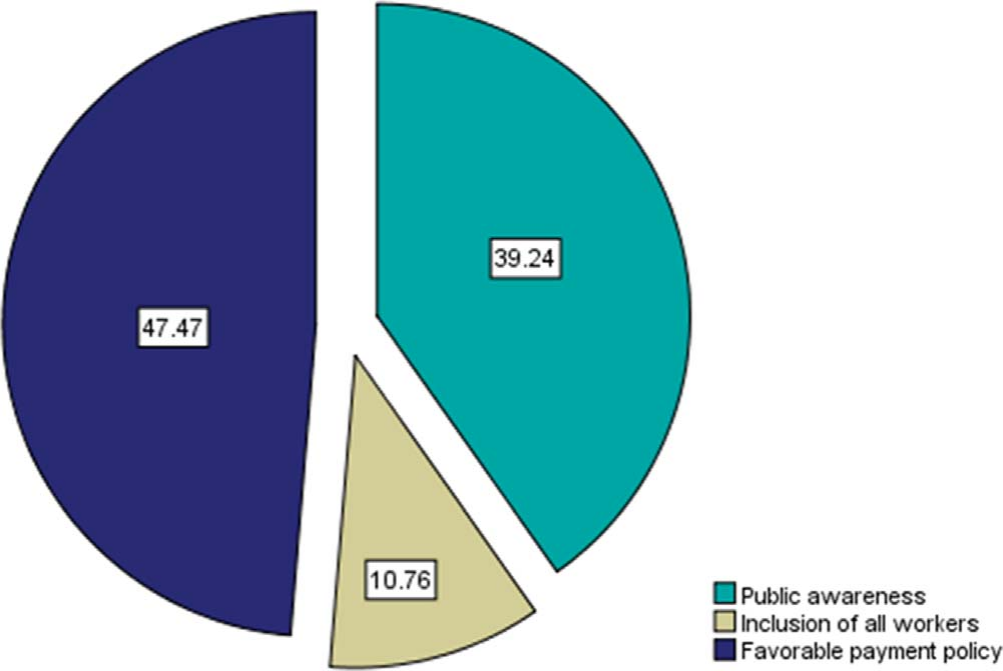
Public opinion on improvement strategies for social health insurance administration.

**Fig. 4:**
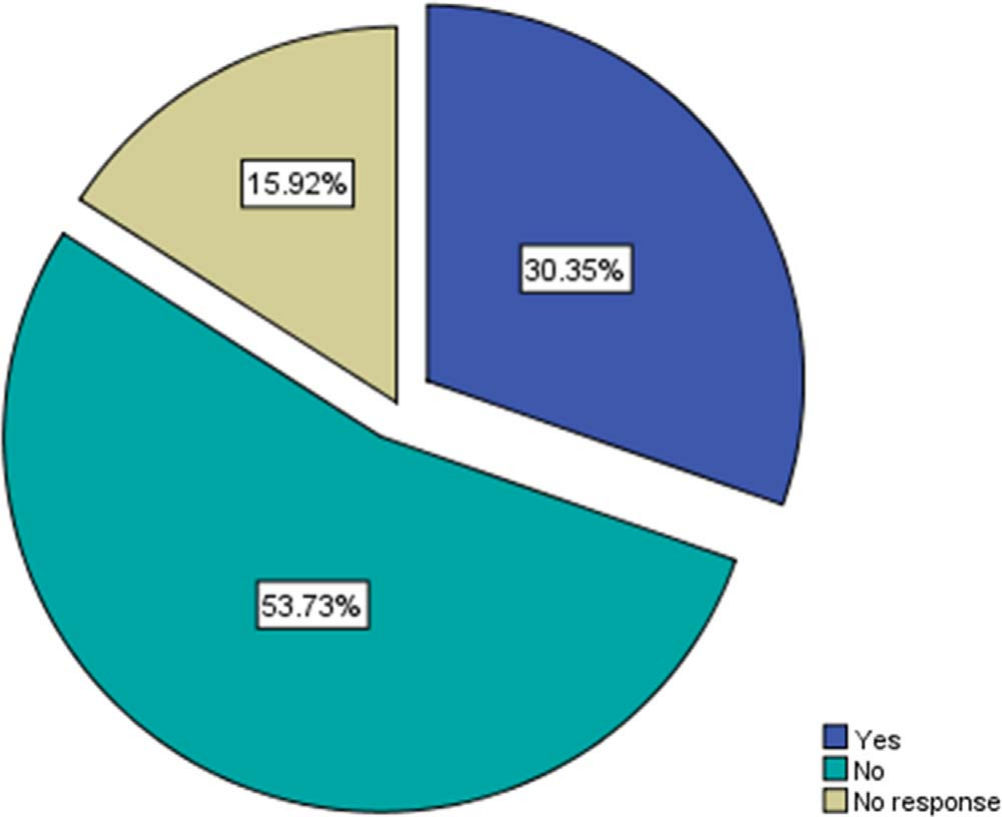
Respondents׳ awareness of different health insurance schemes in Nigeria.

Table 1 shows the age distribution, subscription rate, quality of health of participants and the proportions in different socio-economic classes. Fig. 1, Fig. 2, Fig. 3, Fig. 4 show the average medical cost, government health schemes, improvement suggestions and level of awareness of respondents, respectively (Table 2).

**Table 1:** Quality of health and insurance subscription in different socio-economic classes based on valid responses.

**Parameter**	**Socio-economic classes based on income**						
	**Unemployed freq. (%)**			**High income freq. (%)**	**Uncategorized freq. (%)**	**Ave. income freq. (%)**	**Min. income freq. (%)**
All	25 (12.4)			60 (29.8)	8 (3.9)	67 (33.3)	41 (20.3)
Age range (years)							
15–18		6 (18)		4 (12)	3 (9)	7 (21)	13 (39)
19–45		14 (10)		43 (32)	5 (4)	53 (39)	20 (15)
>45		5 (15)		13 (39)	0	7 (21)	8 (24)
Quality of health							
Good health		24 (16)		46 (31)	5 (3)	43 (29)	30 (20)
Poor health		0		0	0	3 (50)	3 (50)
Fair health		1 (1)		14 (32)	2 (5)	21 (48)	6 (14)
Medical condition		1 (2)		18 (35)	1 (2)	20 (39)	11 (22)
Insurance subscription							
Yes		9 (12)		22 (29)	6 (8)	28 (36)	12 (16)
No		14 (12)		34 (30)	2 (2)	36 (31)	29 (25)
Employer/parent		14 (15)		26 (29)	1 (1)	35 (38)	15 (16)
Privately purchased		1 (4)		8 (31)	2 (8)	8 (31)	7 (27)
Ill Dependants		0		21 (33)	1 (2)	32 (51)	9 (14)
100% coverage		4 (25)		2 (13)	3 (19)	6 (38)	1 (6)
50% coverage		0		12 (52)	1 (4)	6 (26)	4 (17)
<50% coverage		9 (10)		28 (33)	3 (3)	31 (36)	15 (17)
Health coverage abroad		4 (11)		14 (38)	0	16 (43)	3 (8)
No coverage abroad		17 (15)		33 (28)	0	43 (37)	24 (21)
Service delivery							
Satisfactory insurance			9 (12)	27 (36)	1 (1)	26 (35)	11 (15)
Good healthcare			10 (13)	22 (29)	1 (1)	32 (42)	11 (14)
Poor healthcare			9 (15)	25 (40)	3 (5)	13 (21)	12 (19)

**Table 2:** Socio-economic class of employed respondents, insurance subscription and their wellbeing.

**Indicators**	**Total (*N*)**	a**Frequency in minimum income economic class (%)**
Age (years)		
< 45	28	8 (28.5)
> 45	140	33 (23.5)
Health status		
Good	119	30 (25.2)
Fair/Poor	47	9 (19.1)
Chronic medical condition		
Yes	49	11 (22.4)
No	109	27 (24.7)
Insurance subscription		
Yes	59	9 (15.2)
No	102	32 (31.3)
Insurance coverage		
100% coverage	9	1 (11)
<50% coverage	96	19 (19.7)
Quality of health care		
Unsatisfactory	43	9 (20.9)
Satisfactory	75	15 (20)

a Relative to the high and average income socio-economic classes.
